# Drone-assisted time-varying magnetic field analysis for fault diagnosis in grounding grids

**DOI:** 10.1371/journal.pone.0325845

**Published:** 2025-06-17

**Authors:** Aamir Qamar, Zahoor Uddin

**Affiliations:** Department of Electrical Engineering, COMSATS University Islamabd, Wah Campus, Wah, Punjab, Pakistan; Institute of Aviation Engineering and Technology, EGYPT

## Abstract

Grounding grids are essential for ensuring the safety of power substations, but their performance can degrade due to corrosion, fractures, or other faults. Traditional fault diagnosis methods are time-consuming, labor-intensive, and require physical access to substations, posing safety risks. This paper introduces a drone-based approach for magnetic field sensing to diagnose grounding grid faults, significantly reducing operational risks and improving efficiency. However, the movement of the drone introduces time-varying electromagnetic interference (EMI) from substation equipment and the drone itself, complicating the isolation of grounding grid signals. To address this problem, we propose a time-varying un-mixing technique combined with the Fast Independent Component Analysis (FastICA) algorithm to effectively suppress the EMI and extract the grounding grid signals. Simulation results demonstrate the efficacy of the proposed technique in separating grounding grid signals under time-varying conditions, outperforming the FastICA algorithm by 96.36% and the Independent Vector Analysis (IVA) by 41.17% at a block length of 4000 and $\Gamma_{\Delta k}=0.05$. These results highlight the robustness and applicability of the proposed approach for real-world grounding grid fault diagnosis, ensuring accuracy and safety in EMI-rich environments. However, the performance of the proposed technique degrades at higher values of $\Gamma_{\Delta k}$, which represents the speed of the flying drone.

## Introduction

The grounding grid is a mesh of bare conductors installed in power substations beneath the earth’s surface. Its primary function is to safely discharge hazardous currents into the earth during lightning strikes and short circuits [1]. Grounding grids are typically constructed from materials such as copper, steel, aluminum, or galvanized steel. However, moisture and air gaps in the soil can cause corrosion and, in extreme cases, result in fractures within the grounding grid. This deterioration adversely affects the performance of the grounding grid and poses severe threats to the safety of equipment and operators working in the substation [2]. Consequently, researchers have developed various methods over time to periodically monitor the health of grounding grids and diagnose any faults.

Among the various grounding grid fault diagnosis methods, magnetic field sensing techniques are considered the most effective. These include the gradient electromagnetic method [3] and the static fields method [4]. Other methods are the electromagnetic method [5] and the quotient method [6]. Additionally, the electromagnetic and deep learning method [7] is also used, among others. Magnetic field sensing is fundamental to all these methods. However, using raw measured data is not feasible, as it deteriorates the results due to interference from the surrounding magnetic fields generated by various equipment in the substation. Therefore, suppressing the surrounding electromagnetic interference (EMI) or isolating the grounding grid signal from the electromagnetic environment of the substation is crucial to ensure the accuracy of electromagnetic methods for grounding grid fault diagnosis. The Blind Source Separation (BSS) techniques are used in the literature to isolate the grounding grid signal from substation EMI. For instance, the authors in [8] proposed the Independent Component Analysis (ICA), while [3] introduced the Independent Vector Analysis (IVA) to address this challenge. Additionally, magnetic field measurements using conventional methods are time-consuming, require physical access to the substation, and pose safety risks to operators.

The integration of modern technologies into today’s small-sized drones makes them suitable for various applications, including agriculture [9], archaeology [10], remote sensing [11], navigation [12], environmental monitoring [13,14], detecting unwanted drones [15], and the inspection of electrical substations [16]. The authors in [17] proposed end-to-end framework for archaeological sites detection using autonomous service drones. In [18], a navigation system for an autonomous drone was developed for delivering items based on the Global Navigation Satellite System (GNSS). One of the research presented a new method for drone control based on Membrane computing (P systems) [19]. In this paper, the authors introduce a drone-based concept of magnetic field sensing for grounding grid fault diagnosis. This approach minimizes the need for physical access to substations. It also enhances the quality and efficiency of asset management while reducing the associated growth in resource requirements and costs. Additionally, it minimizes safety risks for operators, as the use of drones significantly limits their direct contact with the power substation. However, using drones for magnetic field sensing further amplifies the effects of EMI and complicates grounding grid signal isolation, as drones introduce a time-varying scenario for BSS methods.

**Fig 1:**
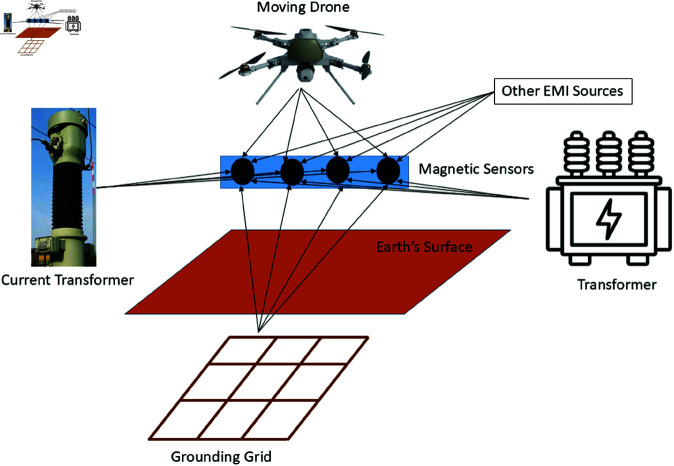
Drone-based magnetic field sensing for grounding grid fault diagnosis.

To the best of our knowledge, this is the first paper in the literature to discuss the application of drones in grounding grid fault diagnosis. Fig 1 illustrates the scenario of magnetic field sensing for this purpose. In the figure, a grounding grid is buried in the soil, and a small drone equipped with magnetic sensors flies at an intermediate speed over the grid to measure its magnetic field. The measured magnetic field consists of the grounding grid’s source signal combined with various EMI signals. These various EMI signals include the drone’s magnetic field, the transformer’s magnetic field, the instrument transformer’s magnetic field, and any other EMI sources located nearby. However, only the grounding grid’s magnetic field (source signal) is required for further analysis to diagnose grounding grid faults. To suppress the unwanted EMI signals, Independent Component Analysis (ICA) is utilized in [20] to unmix the sensed mixed signals. However, in the proposed case, the drone is moving, and the measurements are time-varying, which makes it difficult for ICA to properly separate the mixed signals. To address this issue, we developed a time-varying unmixing technique combined with ICA. This approach effectively separates the sensed mixed signals for further processing and fault diagnosis in the grounding grid.Fig 1.Drone-based magnetic field sensing for grounding grid fault diagnosis.

The main contributions of this research are highlighted below:

- The proposed approach resolves challenges associated with traditional magnetic field measurement techniques, such as being time-consuming, labor-intensive, and risky for operators.
- A time-varying un-mixing technique combined with Independent Component Analysis (ICA) is developed to address the challenges posed by drone-induced time-varying measurements and electromagnetic interference (EMI).
- The proposed drone-based solution reduces the need for physical access to substations, enhancing operational safety by limiting direct contact with power substation environments.
- The paper contributes to overcoming the complexities of grounding grid signal isolation in EMI-rich environments by effectively separating mixed signals for accurate fault diagnosis.

Despite these advancements, the real-world deployment of the proposed approach poses several challenges. These include, proximity of high voltage equipment that can induce eddy currents in the drone’s electronics, leading to failures and crashes. During strong winds, precise control of the drone is required to maintain stability during flight. Real-time sensor data transmission or data acquisition capability is crucial for accurate processing. The former requires robust communication protocols, while the latter needs a data storage facility. Additionally, there may be a drift in sensor readings due to temperature variations.

## System framework

**Fig 2:**
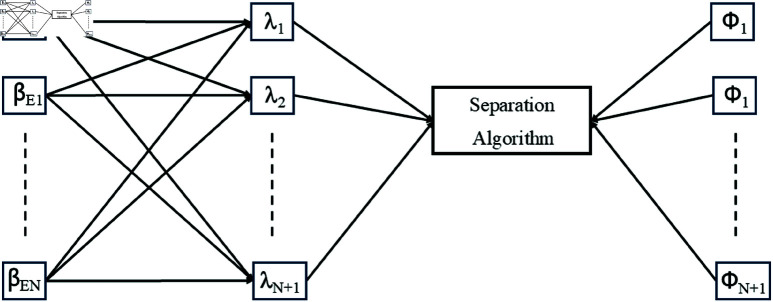
System model of the proposed drone-based magnetic sensing approach for grounding grid fault diagnosis.

This section presents the magnetic field signal of the grounding grid and the interfering signals within the ICA system model. The scenario shown in Fig 1 is re-generated in Fig 2, where a small drone equipped with magnetic sensors measures the grid’s signals while flying at an intermediate speed. The recorded signals consist of the grounding grid’s magnetic field combined with other EMI sources. The proposed technique is used to isolate the grid’s signal, and the system model is described in detail in this section.

For a clearer understanding and generalization of the proposed model, Fig 2 shows N+1 sources, including the grounding grid and EMI sources, recorded through N+1 sensors. The grounding grid signal is represented by $\beta_{G}$ and the EMI sources are labeled as $\beta_{E1},\beta_{E2},\ldots ,\beta_{EN}$, as shown in Fig 2. Each source signal has a block length of *L*, such that $\beta_{EN}=[\beta_{EN1},\beta_{EN2},\ldots ,\beta_{ENL}]$. The magnetic sensors measure mixtures of magnetic fields from the sources. The recorded mixed signals are represented as $\lambda_{1},\lambda_{2},\ldots ,\lambda_{N+1}$. Once the data is sensed, the proposed algorithm processes the mixed signals to perform un-mixing, as illustrated in Fig 2. The resulting unmixed signals are denoted as $\Phi_{1},\Phi_{2},\ldots ,\Phi_{N+1}$. Mathematically, the mixed signals can be expressed as:

λ=Ψβ (1)

Here, λ denotes the (N+1)×L mixed data matrix, Ψ represents the (N+1)×(N+1) mixing matrix, and β corresponds to the (N+1)×L source data matrix. The literature explores various mixing models, including constant mixing, quasi-stationary mixing, ill-conditioned mixing, instantaneous mixing, convolutive mixing, time-varying mixing, non-linear mixing, and under-complete and over-complete mixing [12]. In time-varying scenarios, the mixing coefficients vary within a single processing data block. Under such conditions, (1) can be reformulated as follows:

λ=Ψβ+Δβ (2)

Here, Δβ is referred to as the error signal, and the objective is to account for this parameter during ICA signal processing. The updated mixing matrix, $\Psi^{\prime }$, can now be expressed as:

$$\Psi^{\prime }=\Psi +\Delta$$ (3)

The existing literature mainly assumes small variations in the mixing process, i.e., Δ is negligible, or the length of the processing data block is reduced for negligible variations in the mixing process. Time-varying mixing in the data processing blocks poses a significant challenge when the two aforementioned assumptions no longer hold. Moreover, the separated estimated signals can be expressed as:

Φ=Σλ (4)

Here, Σ is called the un-mixing matrix.

## Adaptive EMI suppression in drone-based magnetic sensing

**Fig 3:**
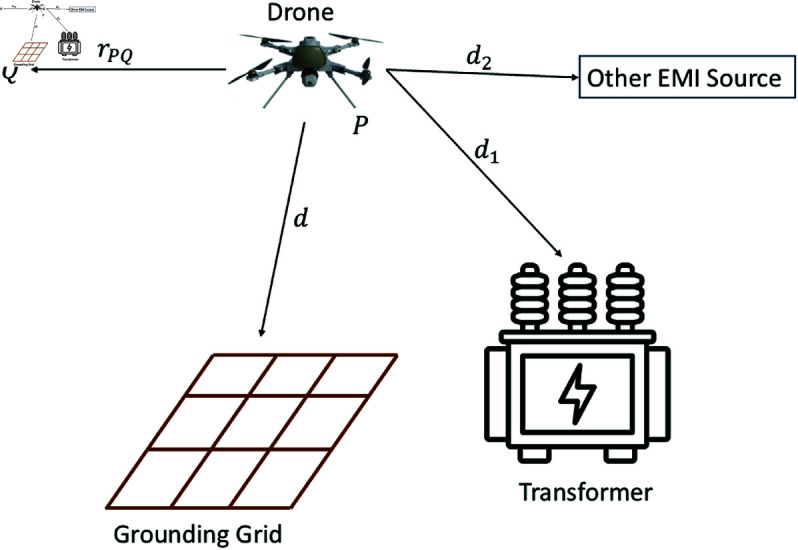
Drone position while flying over the grounding grid.

This section presents the proposed time-varying EMI suppression technique during drone-based magnetic sensing for grounding grid fault diagnosis. A small drone flies over the grid to measure the grounding grid’s magnetic field. Fig 3 is utilized to clearly illustrate the drone’s location while it flies over the grid.

As observed from Fig 3, the drone is initially positioned at *P*, with the grounding grid at a distance *d*, the transformer at a distance *d*_1_, and another EMI source at a distance *d*_2_. At this position, the magnetic sensing unit installed on the drone detects a mixture of all signals. It is important to note that signal attenuation depends on the distance from the magnetic sensing unit. At point *P*, the mixing matrix for *N* + 1 number of sources can be written as follows:

$$\Psi =[\begin{array}{cccc} \psi_{11} & \psi_{12} & \cdots & \psi_{1(N+1)}\\ \psi_{21} & \psi_{22} & \cdots & \psi_{2(N+1)}\\ \vdots & \vdots & \ddots & \vdots \\ \psi_{(N+1)1} & \psi_{(N+1)2} & \cdots & \psi_{\left(N+1)(N+1\right)} \end{array}]$$ (5)

Here, all $\psi_{ij}$ are the mixing coefficients for all i,j=1,2,…N+1. When the drone moves a distance *r*_*PQ*_ from *P* to *Q*, the distances vary, causing changes in the mixing coefficients. The new mixing coefficients are then expressed as:

$${\psi^{n}}_{ij}=\psi_{ij}+\Delta_{ij}$$ (6)

The newly obtained mixing matrix is represented as follows:

$$\Psi^{\prime }=[\begin{array}{cccc} \psi_{11}+\Delta_{11} & \psi_{12}+\Delta_{12} & \cdots & \psi_{1(N+1)}+\Delta_{1(N+1)}\\ \psi_{21}+\Delta_{21} & \psi_{22}+\Delta_{22} & \cdots & \psi_{2(N+1)}+\Delta_{2(N+1)}\\ \vdots & \vdots & \ddots & \vdots \\ \psi_{(N+1)1}+\Delta_{((N+1)1} & \psi_{(N+1)2}+\Delta_{(N+1)2} & \cdots & \psi_{\left(N+1)(N+1\right)}+\Delta_{\left(N+1)(N+1\right)} \end{array}]$$ (7)

In time-varying scenarios, the mixing coefficients vary within the processing data blocks, preventing the ICA algorithm from effectively separating the mixed recorded signals.

The proposed drone-based magnetic field sensing and separation technique performs well as shown in the simulation section and explained mathematically in the rest of this section. The source data matrix β contains the grounding grid signal along with the interfering EMI signals. During a single flight of the recording drone, a large data set λ is recorded. After recording the large data set, split it into multiple *K* number of small data sets as expressed by $\lambda_{1},\lambda_{2},\ldots ,\lambda_{K}$, where k=1,2,…,K. The splitting criteria is explained later in this section. It is also important to note that the mixing matrices associated with $\lambda_{1},\lambda_{2},\ldots ,\lambda_{K}$ are $\Psi_{1},\Psi_{2},\ldots ,\Psi_{K}$. After computing $\Psi_{k}$, through the ICA algorithm the $\Delta_{k}$ can be expressed as follows:

$$\Delta_{k}=\Psi_{k+1}-\Psi_{k}=[\begin{array}{cccc} \Delta_{11} & \Delta_{12} & \cdots & \Delta_{1(N+1)}\\ \Delta_{21} & \Delta_{22} & \cdots & \Delta_{2(N+1)}\\ \vdots & \vdots & \ddots & \vdots \\ \Delta_{(N+1)1} & \Delta_{(N+1)2} & \cdots & \Delta_{\left(N+1)(N+1\right)} \end{array}]$$ (8)

Note that $\Psi_{k+1}$ and $\Psi_{k}$ represent mixing matrices associated with the mixed data matrices $\lambda_{k+1}$ and $\lambda_{k}$. It must also be noted that $\Delta_{k}$ shows the variations in the mixing matrices while moving from one point to another point. Once $\Delta_{k}$ is determined, the $\Gamma_{\Delta_{k}}$ can be determined as:

$$\Gamma_{\Delta_{k}}=\sum_{i=1}^{N+1}\sum_{j=1}^{N+1}|\Delta_{ij}|$$ (9)

The $\Gamma_{\Delta_{k}}$ measures variations prediction in the channel. In the channel, high $\Gamma_{\Delta_{k}}$ shows large variations and vice-versa. Eventually, the condition checked is expressed as:

$$\Gamma_{\Delta_{k}+1}\leq \Gamma_{\Delta k}$$ (10)

If the above condition holds, choose another data set $\lambda_{k+1}$. Otherwise, reduce the data block length and repeat the separation process. Notably, for quasi-static mixing, increasing the data block length improves results. However, in time-varying scenarios, this approach fails due to significant variations in block length. Furthermore, reducing the processing data block length minimizes variations in the mixing process. Based on these considerations, we reduced the data block length in case of worse performance of the separation algorithm. Finally, select the extracted results $\Phi_{k}$ corresponding to the minimum value of $\Gamma_{\Delta k}$. The overall technique is summarized step by step as follows:

**Algorithm:**

**Initialization:**

a. Set the value of *K* (number of data blocks).

b. Set the value of *L* (initial data block length).

c. Select the maximum number of source signals *N* + 1.

d. Initialize a random mixing matrix of size (N+1)×(N+1).

**Step 1:** Store long dataset λ as λ=Ψβ.

**Step 2:** Compute the total length of dataset λ:

μ=Length(λ).

**Step 3:** Divide dataset λ into *K* blocks:

$\mu^{\prime }=\mu /K$.

Set $\text{Length}(\lambda )=\mu^{\prime }$.

**for** *k* = 1 to *K* **do**

a. Select data block $\lambda_{k}$ from λ.

b. Perform un-mixing using the FastICA algorithm.

c. Store the unmixed dataset $\Phi_{k}$.

d. Compute mixing matrix $\Psi_{k}$ from un-mixing weights $\Sigma_{k}$.

e. Compute variations in mixing matrix:

$\Delta_{k}=\Psi_{k+1}-\Psi_{k}$.

f. Calculate channel variation measure:

$\Gamma_{\Delta_{k}}=\sum_{i=1}^{N+1}\sum_{j=1}^{N+1}|\Delta_{ij}|$.

g. Store the value of $\Gamma_{\Delta_{k}}$.

**end for**

**Step 4:** Check condition:

**if** $\Gamma_{\Delta_{k+1}}\leq \Gamma_{\Delta_{k}}$ **then**

Increment *k* and proceed to the next data block.

**else**

a. Increment *k*.

b. Decrease data block length *L* by adjusting *K*: *K* = *K* + 1.

c. Recalculate and repeat steps.

**end if**

**Step 5:** Select $\Phi_{k}$ corresponding to the minimum $\Gamma_{\Delta_{k}}$.

**Terminate Algorithm.**

In the above discussion, (8) represents changes in the mixing matrices as the drone flies from one point to another, capturing channel variations during movement. The results obtained in (8) are in the form of different matrices, so (9) is developed to combine these results into single values to predict variations in the channel. Finally, the results in (9) are compared to determine the best possible outcome, with the selection criteria given in (10). The physical significance of (9) and (10) in EMI suppression is to predict channel variations through (9) and obtain the best possible separated signals through (10). If (10) still fails to produce acceptable results, an alternative approach is to process the reduced-length mixed recorded signals for separation, where reduced length refers to small variations in the channel.

As a concluding remark, we claim that high-quality results lead to better suppression of unwanted EMI and, consequently, improved fault diagnosis. It must also be noted that EMI suppression is a fundamental step in the proposed concept of drone-based magnetic field sensing for grounding grid fault diagnosis, as fault diagnosis is not possible without EMI suppression in a practical scenario.

The proposed time-varying EMI suppression technique effectively enhances the accuracy of drone-based magnetic sensing for grounding grid fault diagnosis. By leveraging the spatial variation of magnetic signals during drone flight and strategically segmenting the recorded dataset into smaller blocks, the technique accounts for time-varying mixing coefficients. The application of the ICA algorithm, combined with the computation of variation metrics $\Gamma_{\Delta_{k}}$, enables the accurate separation of mixed signals, even in dynamic environments with significant interference.

## Simulations

The effectiveness of the proposed grounding grid magnetic field sensing and EMI suppression technique using a small-size drone is evaluated in this section. The overall scenario becomes time-varying due to the drone’s movement. Notably, Fig 1 depicts the practical scenario of the proposed technique. Similarly, Fig 3 illustrates the movement of the flying drone from one specific point to another, showing how the the mixing coefficients change with its position.

**Fig 4:**
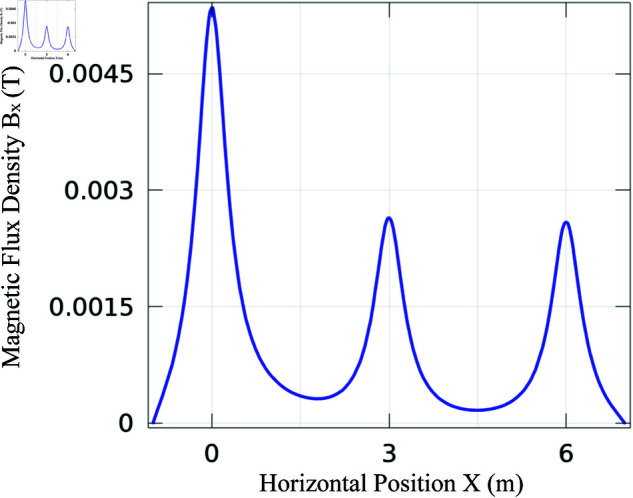
Grounding grid (source signal) magnetic field [12].

**Fig 5:**
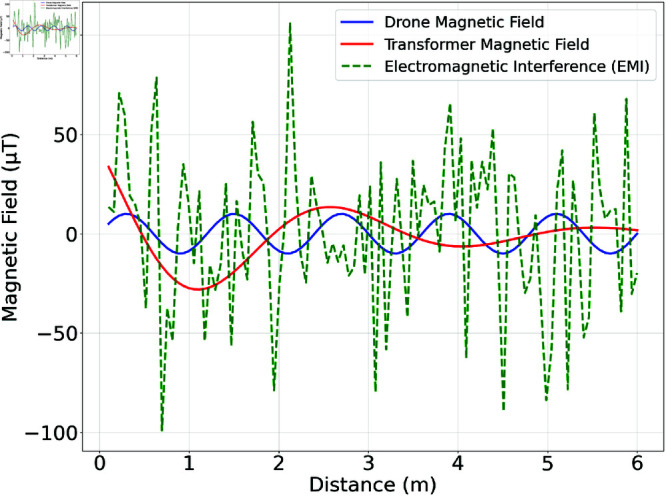
Magnetic fields from the drone and transformer, along with other EMI. The transformer magnetic field decays with respect to the distance as the drone magnetic field is constant.

A performance evaluation was conducted using Monte Carlo simulations, which were performed with MATLAB such that the algorithm is iterated 1000 times and average value of the results is considered. We utilized the grounding grid signal in combination with the drone, transformer and any other EMI signals. The grounding grid (source signal) magnetic field is illustrated in Fig 4. This magnetic field is taken from [4], corresponding to Fig 3 of the article. It is obtained from the magnetic field sensing of the grounding grid illustrated in Fig 2a of the same article. Three main peaks are observed, indicating the locations of the grounding conductors. The magnetic fields of the drone, transformer, and other EMI generated in Python are shown in Fig 5. Here the drone’s magnetic field is modeled as a sinusoidal variation with distance, while keeping its magnitude constant since magnetic sensors are installed on it. Its amplitude is set to 10μT. The transformer’s magnetic field is modeled as an exponentially decaying sinusoidal function as the drone moves away from it, with a maximum amplitude of 50μT and a frequency of 50 Hz, matching power grid frequency. Additionally, the EMI is modeled as randomly generated Gaussian noise with a mean of 0μT and a standard deviation of 40μT. In Monte Carlo simulations, the drone’s position was sampled along a 1D measurement path and at each sampled location a random EMI value was mixed to reflect the fact that EMI varies randomly along the measurement path. This simulates time-varying EMI conditions across the drone’s flight path, ensuring that EMI varies with position and closely reflects real-world conditions. Moreover, there is no frequency mixing and phase shifts among the various sources.

Additionally, the lengths of the processing data blocks used in these simulations range from 1,000 to 10,000 samples. The performance evaluation criterion employed is the signal-to-interference ratio (SIR) [15,21]. SIR measures how distinct a separated signal is from other signals, essentially indicating the quality of the extracted signal. SIR in decibels (dB) for a single data block is expressed as follows:

$$SIR_{\left(\text{dB}\right)}=10\log\left(\frac{1}{L}\sum_{n=1}^{L}\frac{\beta (n{)}^{2}}{(\beta (n)-\Phi (n){)}^{2}}\right)$$ (11)

Quasi-static and time-varying scenarios are utilized in these simulations. In case of time-varying scenario the mixing matrix varies within a single data set and expressed as follows:

$$\Psi^{\prime }=[\begin{array}{cccc} \psi_{11}+\Delta_{11} & \psi_{12}+\Delta_{12} & \psi_{13}+\Delta_{13} & \psi_{14}+\Delta_{14}\\ \psi_{21}+\Delta_{21} & \psi_{22}+\Delta_{22} & \psi_{23}+\Delta_{23} & \psi_{24}+\Delta_{24}\\ \psi_{31}+\Delta_{31} & \psi_{32}+\Delta_{32} & \psi_{33}+\Delta_{33} & \psi_{34}+\Delta_{34}\\ \psi_{41}+\Delta_{41} & \psi_{42}+\Delta_{42} & \psi_{43}+\Delta_{43} & \psi_{44}+\Delta_{44} \end{array}]$$ (12)

**Fig 6:**
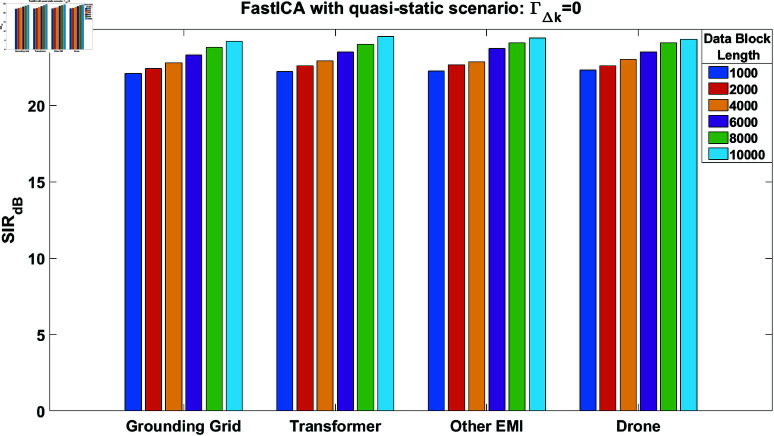
SIR performance of the FastICA algorithm in case of quasi-static condition i.e., $\Gamma_{\Delta k}$ = 0, for all the source estimated signals.

**Fig 7:**
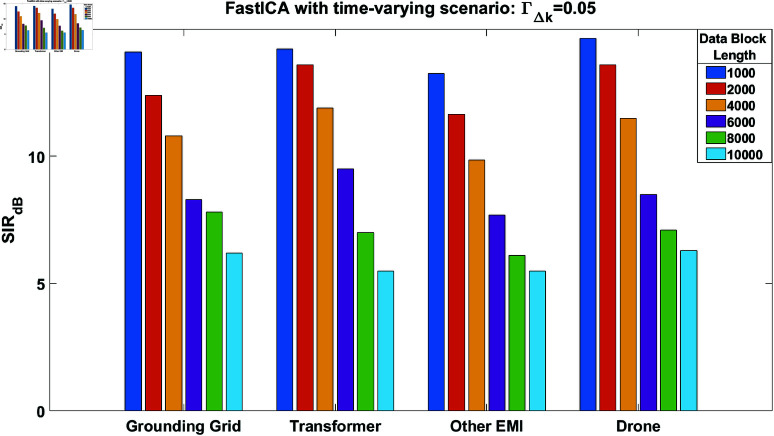
SIR performance of the FastICA algorithm in case of time-varying condition at $\Gamma_{\Delta k}$ = 0.05, for all the source estimated signals.

The SIR performance of the FastICA algorithm [15] was evaluated first for the quasi-static case i.e, $\Gamma_{\Delta k}=0$, while utilizing generated source signals in MATLAB. Since the FastICA algorithm typically converges in a few iterations, we set the maximum number of iterations to 200 in our simulations. Additionally, the stopping criterion used is $\|\sigma_{\text{new}}\;-\;\sigma_{\text{old}}\|\leq 10^{-6}$, where 10^−6^ is the epsilon value. The $\sigma_{\text{new}}$ and $\sigma_{\text{old}}$ represent column vectors of the un-mixing matrix Σ obtained through the FastICA algorithm. The data block lengths range from 1,000 to 10,000 samples with a signal-to-noise ratio (SNR) of 20 dB. Fig 6 illustrates the SIR performance of all four source signals, where the SIR values increase as the data block length increases. In addition to this, we also evaluate the SIR performance of the FastICA algorithm [22] in time-varying scenario as shown in Fig 7. In these simulations, we kept the $\Gamma_{\Delta k}=0.05$. From Fig 7 it can be clearly observed that performance of the algorithm degrades with increase in block length. In case of increased block lengths, variations increase within the processing data blocks. At a block length of 10,000 samples, the SIR performance of the grounding grid signals in Fig 6 is 24, while in Fig 7 it is 6. The results obtained in Fig 7 are not considerable, indicating the need for a performance improvement technique in a time-varying scenario.

**Fig 8:**
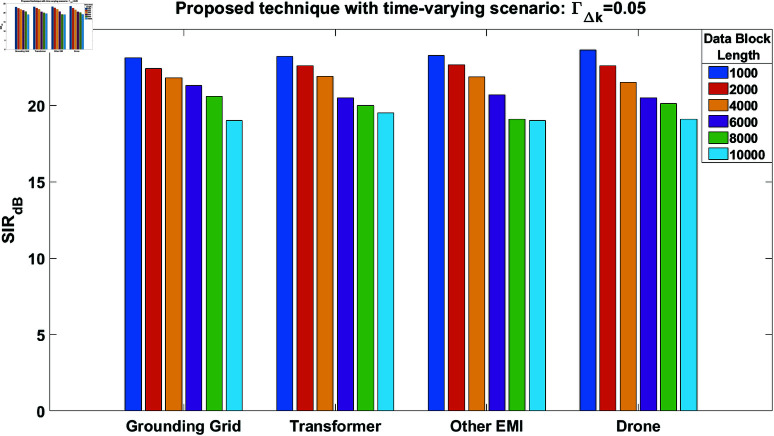
SIR performance of the proposed technique in case of time-varying scenario at $\Gamma_{\Delta k}$ = 0.05, for all the separated source signals.

The SIR performance of the proposed technique is next evaluated for $\Gamma_{\Delta k}=0.05$. The results are summarized in Fig 8 for all the source signals and data blocks. From this figure, a clear performance improvement can be observed compared to Fig 7. It should also be noted that the performance degradation in Fig 8 with an increased length of the processing data block is evident, primarily due to increased variations within the data blocks.

**Fig 9:**
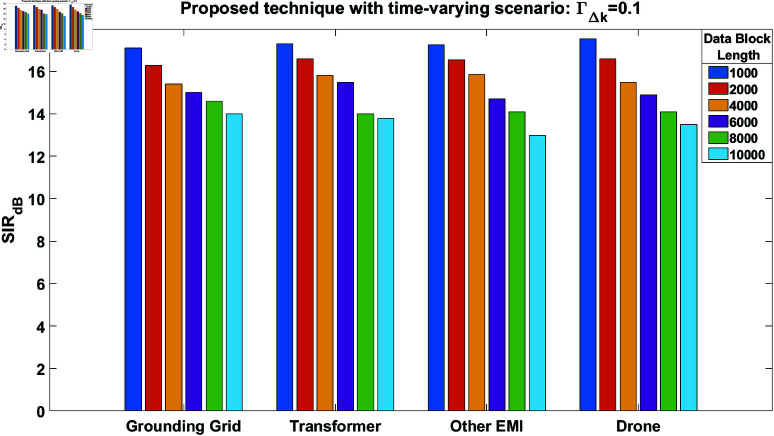
SIR performance of the proposed technique in case of time-varying scenario at $\Gamma_{\Delta k}$ = 0.1, for all the source signals.

**Fig 10:**
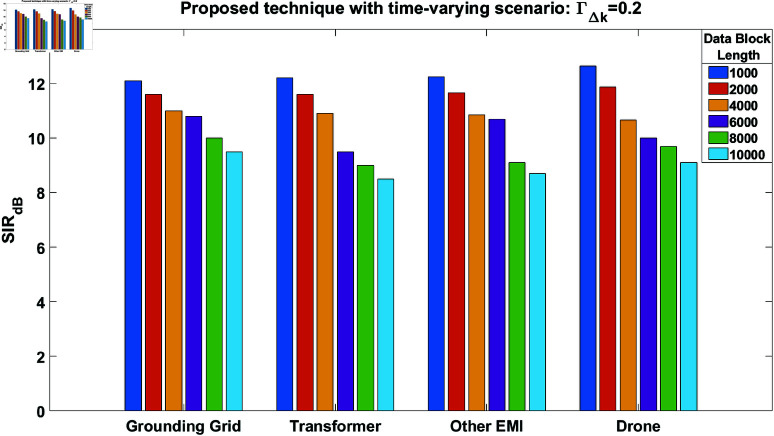
SIR performance of the proposed technique in case of time-varying scenario at $\Gamma_{\Delta k}$ = 0.2, for all the separated source signals.

Furthermore, the time-varying scenario is again considered while utilizing the proposed technique with $\Gamma_{\Delta k}=0.1$. The results are shown in Fig 9, which still demonstrate good-quality performance even with increased time variations in the mixing channel. In another simulation setup, we consider $\Gamma_{\Delta k}=0.2$ and perform the simulations again. The results are presented in Fig 10. This figure also shows acceptable performance, despite further increases in time variations within the channel. However, with a further increase in $\Gamma_{\Delta k}$, the results become unacceptable, which is why the value of $\Gamma_{\Delta k}$ is limited to 0.2. Furthermore, the simulation results are presented in Table 1 to clearly demonstrate the performance improvement of the proposed technique in a time-varying scenario. This table compares the performance of the proposed technique with FastICA for different block lengths and values of $\Gamma_{\Delta k}$.

**Table 1:** Comparison of the FastICA and the proposed technique in terms of SIR for quasi-static and time-varying conditions utilizing the grounding grid signals.

S.No.	L	FastICA at $\Gamma_{\Delta \text{k}}$ = 0	FastICA at $\Gamma_{\Delta \text{k}}$ = 0.05	Proposed at $\Gamma_{\Delta \text{k}}$ = 0.05	Proposed at $\Gamma_{\Delta \text{k}}$ = 0.1	Proposed at $\Gamma_{\Delta \text{k}}$ = 0.2
1.	1000	22.2	14.3	22.1	17.5	12
2.	2000	22.4	12.8	22	16.2	11.7
3.	4000	22.7	11	21.6	15.7	11
4.	6000	23.6	8.3	21	15	10.7
5.	8000	24	8	20.8	14.7	10
6.	10000	24.4	6.2	18.5	14	9.5

**Fig 11:**
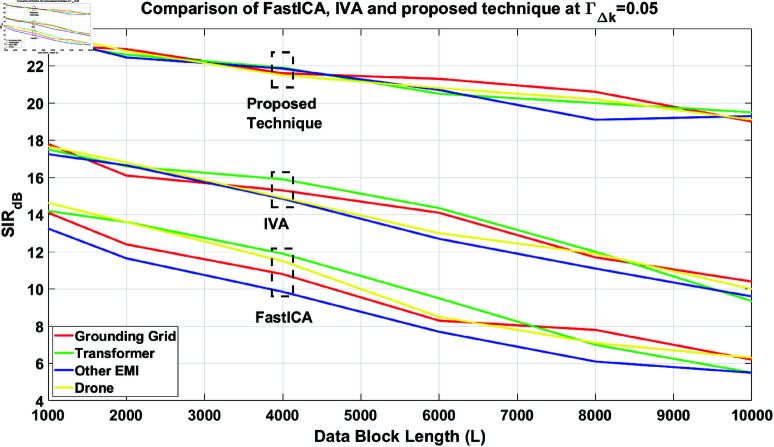
Performance comparison of the FastICA, IVA, and the Proposed technique in time varying scenario at $\Gamma_{\Delta k}$ = 0.05, for all the separated source signals.

Finally, a performance comparison between FastICA, IVA, and the proposed technique is provided in Fig 11, with $\Gamma_{\Delta k}=0.05$. It should be noted that the Gradient (IVA-G) algorithm [21] of the IVA is utilized in these simulations. The figure highlights the superior performance of the proposed technique under a time-varying parameter of 0.05. Although IVA outperforms the FastICA algorithm, the proposed technique remains superior to IVA.

From the figure, it is observed that the proposed technique achieves an SIR of 21.6 dB at a block length of 4000, while IVA achieves 15.3 dB and FastICA achieves 11 dB for the grounding grid (source) signal, thereby outperforming IVA by 41.17% and FastICA by 96.36%. However, the performance of the proposed technique degrades as the value of $\Gamma_{\Delta k}$ increases, where $\Gamma_{\Delta k}$ is a parameter that reflects the speed of the flying drone.

As concluding remarks, we can say that the proposed approach ensures adaptability by dynamically adjusting data block lengths based on performance, optimizing the separation process for both quasi-static and time-varying scenarios. As demonstrated in simulations, the proposed method consistently achieves reliable separation of the grounding grid signal from interfering EMI sources. These results highlight the technique’s robustness and its potential for application in real-world grounding grid fault diagnosis systems.

## Conclusion

The authors of this paper introduced the concept of drone-assisted magnetic field sensing for diagnosing grounding grid faults. Additionally, they proposed ICA based time-varying un-mixing technique that enables effective suppression of the electromagnetic interference (EMI) in dynamic environments. Simulation results demonstrate that the proposed method consistently isolates grounding grid signals with acceptable accuracy, even under challenging time-varying conditions induced by drone movement. The proposed approach significantly enhances the safety and efficiency of grounding grid fault diagnosis by eliminating the need for physical access to substations. It dynamically adjusts data block lengths to ensure adaptability and optimized performance in time-varying scenarios. By addressing the complexities of EMI-rich environments, this work lays the foundation for deploying drone-based fault diagnosis in real-world applications, ensuring robustness, accuracy, and improved operational safety in power substations.
